# A High-Resolution Digital Bathymetric Elevation Model Derived from ICESat-2 for Adam’s Bridge

**DOI:** 10.1038/s41597-024-03550-3

**Published:** 2024-06-27

**Authors:** Giribabu Dandabathula, Rohit Hari, Jayant Sharma, Aryan Sharma, Koushik Ghosh, Niyati Padiyar, Anisha Poonia, Apurba Kumar Bera, Sushil Kumar Srivastav, Prakash Chauhan

**Affiliations:** 1 Regional Remote Sensing Centre - West, National Remote Sensing Centre, Indian Space Research Organisation, Jodhpur, India; 2 Computer Science Department, Jaipur Engineering College and Research Centre (JECRC) University, Jaipur, India; 3 Department of Geography, Panjab University, Chandigarh, India; 4 Amity Institute of Geoinformatics and Remote Sensing, Amity University, NOIDA, India; 5 Regional Centres, National Remote Sensing Centre, Indian Space Research Organisation, New Delhi, India; 6 National Remote Sensing Centre, Indian Space Research Organisation, Hyderabad, India

**Keywords:** Geophysics, Physical oceanography, Hydrology, Limnology

## Abstract

This data descriptor elaborates the details of a high-resolution digital bathymetric elevation model generated for the region, namely, Adam’s Bridge, which encompasses a chain of shoals between Rameswaram Island, off the southeastern coast of Tamil Nadu, India, and Mannar Island, off the northwestern coast of Sri Lanka. The proposed dataset has taken advantage of the photon penetrability in the shallow waters by the green laser of ICESat-2 LiDAR to derive the seabed topography. Seafloor depths from ~0.2 million geolocated photons of ICESat-2 for the study area were accrued and interpolated to generate a 10 m digital bathymetric elevation model. Adam’s Bridge, an isthmus and submerged reefal assemblage in shallow and super-shallow waters, is a feature of scientific curiosity. Our dataset has the potential to enhance the understanding of Adam’s Bridge structure by providing substantial information to reconstruct its evolution.

## Background & Summary

**Fig. 1:**
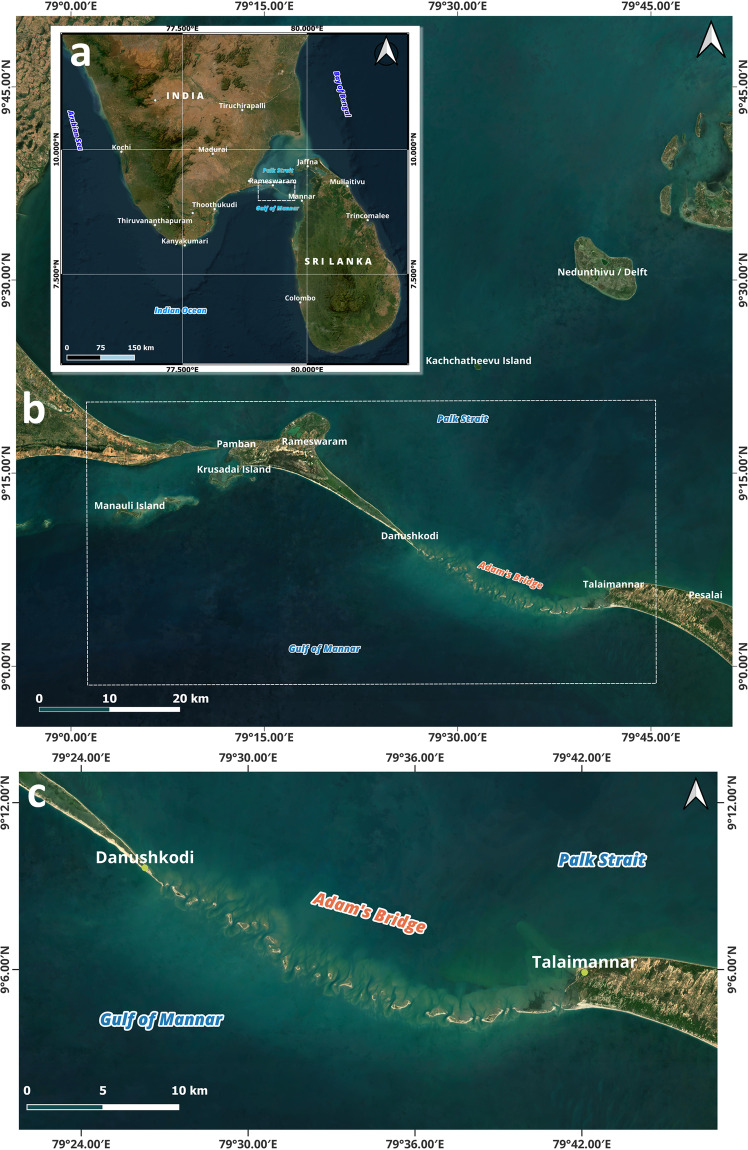
Adam’s Bridge and its surroundings as seen in satellite imagery. **(a)** Map showing southern India and Sri Lanka. **(b)** The extent marked with the white box comprises part of India, including Pamban/Rameswaram Island, with its eastern point named Dhanushkodi, Adam’s Bridge, and Talai Mannar, the western end of Mannar Island, Sri Lanka. **(c)** A zoomed extent showing the exposed sandbanks of Adam’s Bridge. The satellite imagery used in the maps is from the web mapping services of the Sentinel-2 cloudless layer for 2021 by EOX (https://s2maps.eu/ and https://esa.maps.eox.at/).

Adam’s Bridge is an isthmus of length ~30 km connecting Dhanushkodi, the southeastern point of Rameswaram Island, off the southeastern coast of Tamil Nadu, India, and Talai Mannar, the western end of Mannar Island, off the northwestern coast of Sri Lanka^1–11^; the extent along with its surroundings is shown in Fig. 1 comprising high-resolution satellite imagery. The southern part of the Adam’s Bridge has the Gulf of Mannar, an arm of the Indian Ocean, and Palk Strait, an inlet of the Bay of Bengal, in its northern direction.

On this heavily submerged isthmus, loose and fine sandbanks are exposed at irregular intervals above the water level. Generally, these sandbanks exhibit seasonal shifts; moreover, these sandbanks have no presence of rocks^1,2,10^. Most of this isthmus crest (the central position of the submerged ridge) is within the depth of 1 m of the water currents with great rapidity, but in some parts, sudden channels of depth 2-3 m exist; however, this depth is more when the channels are narrower^10^. Studies concerning the material strata through borings done by various surveyors yielded sand up to a depth of 7–9 m, and further depths yielded Holocene conglomerate, beds of limestone, calcareous sandstone, and occasional corals^1,7,8,10,12,13^. Next to this second strata, a layer of sand followed by hard rocks or continuous compact formation was detected^12,13^. Such a type of reefal assemblage is a matter of scientific curiosity, and further understanding of its morphological structure can provide substantial information to reconstruct its evolution.

Besides the interest in the unique geological structure of Adam’s Bridge, right from the 19^th^ century, it has remained at the center of controversies and debates surrounding the reasons for attributing its name and origin (man-made or geological formation)^14–46^. From the ages, with reference to the Hindu mythology called Ramayana, this geographic extent, in most South Asian countries, is referred to as Ram Setu^23–27,46,47^. Major James Rennel (1742–1830), under the capacity of Surveyor General of Bengal for East India Company, labeled this geographic extent as Adam’s Bridge in all the provincial maps that he produced; many European navigators were already referring to this extent by the same name by that time^41,42^. Erstwhile, many committees were appointed to propose a railway line connecting India with Sri Lanka through Adam’s Bridge^45–47^, and also, plans for dredging operations that can enable a shipping canal to reduce the distance and cost of navigation due to circumnavigation around Sri Lanka^14,46–58^. Recently, with the advent of optical satellite imagery, researchers have reported about the exposed parts of Adam’s Bridge, which contains small patch reefs lying irregularly with sand cays^44^.

Bathymetric procedures measure water depth (relative to a reference surface such as sea level) in the oceans, seas, rivers, and lakes, typically representing a topographic seafloor surface, which is a vital parameter for numerous applications in marine and ocean engineering^59–62^. High-resolution bathymetric datasets are the most accessible methods to analyze submerged reef features^63^. Adam’s Bridge is a submerged feature, and its understanding can be advanced using high-resolution bathymetric data. Technologies that generate the bathymetric data include ship-borne single/multi-beam echo sounders, satellite-derived, and aerial/satellite altimeter data^62^. However, bathymetric surveying in shallow and super-shallow waters by conventional ship-borne sonar techniques poses challenges during the data acquisition, and also, the acoustic signals get distorted, impacting the measurement accuracy^64,65^. Moreover, as Adam’s Bridge is mainly submerged in super-shallow waters, ship-borne sounding data within its vicinity is unavailable from any navigational charts.

Satellite-derived bathymetric methods employ empirical methods that usually aid in the completeness of the charts and are not a replacement for acoustic or active remote sensing-based Hydrographic surveys^62,66^. Non-imaging active remote sensing methods like LiDAR can provide highly accurate depth information in shallow waters^67^. As the extent of Adam’s Bridge lies submerged in shallow and super-shallow waters^2,10,68–70^, the preference for using LiDAR data should aid in generating the bathymetric data. However, water clarity is an important parameter that influences the accuracy of the LiDAR data. Water clarity is a physical characteristic defined by how transparent the water is and determined by the depth that light penetrates in water. The more the sediments in the water, the more the light attenuates, i.e., diminished by scattering (changing the direction of propagation) or absorption before reaching the seabed. Thus, data acquisitions from the seasons of less sediment should be considered while generating the bathymetry from the LiDAR sensors.

Free and open accessible global bathymetric data sources include the General Bathymetric Chart of the Oceans (GEBCO), which is available at ~450 m spatial resolution (https://www.gebco.net/), and the Global Multi-Resolution Topography (GMRT), synthesized using ship-based multi-beam data, is available at 100 m spatial resolution (https://www.gmrt.org/). An experiment assessing the performance of the GEBCO bathymetric dataset at the extent of Adam’s Bridge and its surroundings has proven to have erratic fluctuations in the seafloor compared with reference data^71^. In the case of GMRT datasets, multi-beam sonar data is used, which are collected and contributed by various scientific communities. The GMRT science teams combine these contributed datasets into a continuous global compilation. Due to the lack of multi-beam sonar data by any scientific institution near Adam’s Bridge and its surroundings, the current form of GMRT bathymetric data consists of only hypothetical values in the said extent^72^. Notably, due to their coarser resolution, these open-access bathymetric datasets may not help depict the intricate details of Adam’s Bridge (as will be discussed later in the technical validation section of this report).

Ice, Cloud and land Elevation Satellite-2 (ICESat-2)^73^ is regarded as a revolutionary space-borne altimeter due to its applicability for many applications in Earth sciences^74^. Various data products from ICESat-2 contain pertinent information to essay the Earth’s surface variations^75^. Besides using the ICESat-2 data for understanding cryosphere, land, and canopy, researchers have investigated the applicability of ICESat-2 geolocated photons for bathymetric studies and concluded that detecting seafloor depths of up to ~40 m in shallow waters is possible abiding by certain prerequisite conditions^76^. Studies that validated the performance of ICESat-2 photons for deriving water depths have reported accuracy in the range of 0.20 m to 0.89 m^76–80^; these results are highly significant in terms of accuracy and, by far, the best from the current operational space-borne active LiDAR sensors.

The dataset described in this article gives details of the Digital Bathymetric Elevation Model (DBEM) generated using ICESat-2 geolocated photons for the extent of Adam’s Bridge. At the outset, this DBEM has taken advantage of photon penetration into the shallow waters by the green laser of ICESat-2, which enabled the amicability to derive the seafloor depth for shallow waters; using this as a cue, we have accrued depths from ~0.2 million geolocated photons for the study area and interpolated to generate a 10 m DBEM. The data has the potential usage to understand the intricate details of the physical structure of Adam’s Bridge. The proposed DBEM can be integrated into the models to understand Adam’s Bridge’s morphology, surficial-sediment characterization, and wave dynamics originating from the Gulf of Mannar and the Palk Strait.

## Methods

### Study area

The extent of the study area for which the proposed dataset was generated is shown in Fig. 1b and marked with a white box. The presence of the Adam’s Bridge, as seen in optical satellite data, is shown in Fig. 1c,where mostly, in its entire form, is a submerged ridge with occasional exposure to sand cays at irregular intervals. From the tip of Dhanushkodi, Adam’s Bridge’s general trending long axis is oriented from west-northwest to east-southeast in secondary intercardinal directions, entirely perpendicular to the predominant wave approach directions of both the Gulf of Mannar and the Palk Strait. The general direction of Adam’s Bridge for the first 17 km of its length is about east-south-east; later, the direction is gradually curved towards the north of the east and finally touches Talaimannar Island at its east. The study area includes neighbouring stretches of Adam’s Bridge like Rameswaram Island (on the Indian side), part of Mannar Island (on the Sri Lankan side), and the surrounding waters of the Gulf of Mannar and Palk Strait; the rationale for extending the study area beyond the core area of interest, i.e., Adam’s Bridge, is to accommodate the relationship between the location of interest and the distance to surrounding sample points during the digital seafloor surface generation.

### Data resources and methodology

**Fig. 2:**
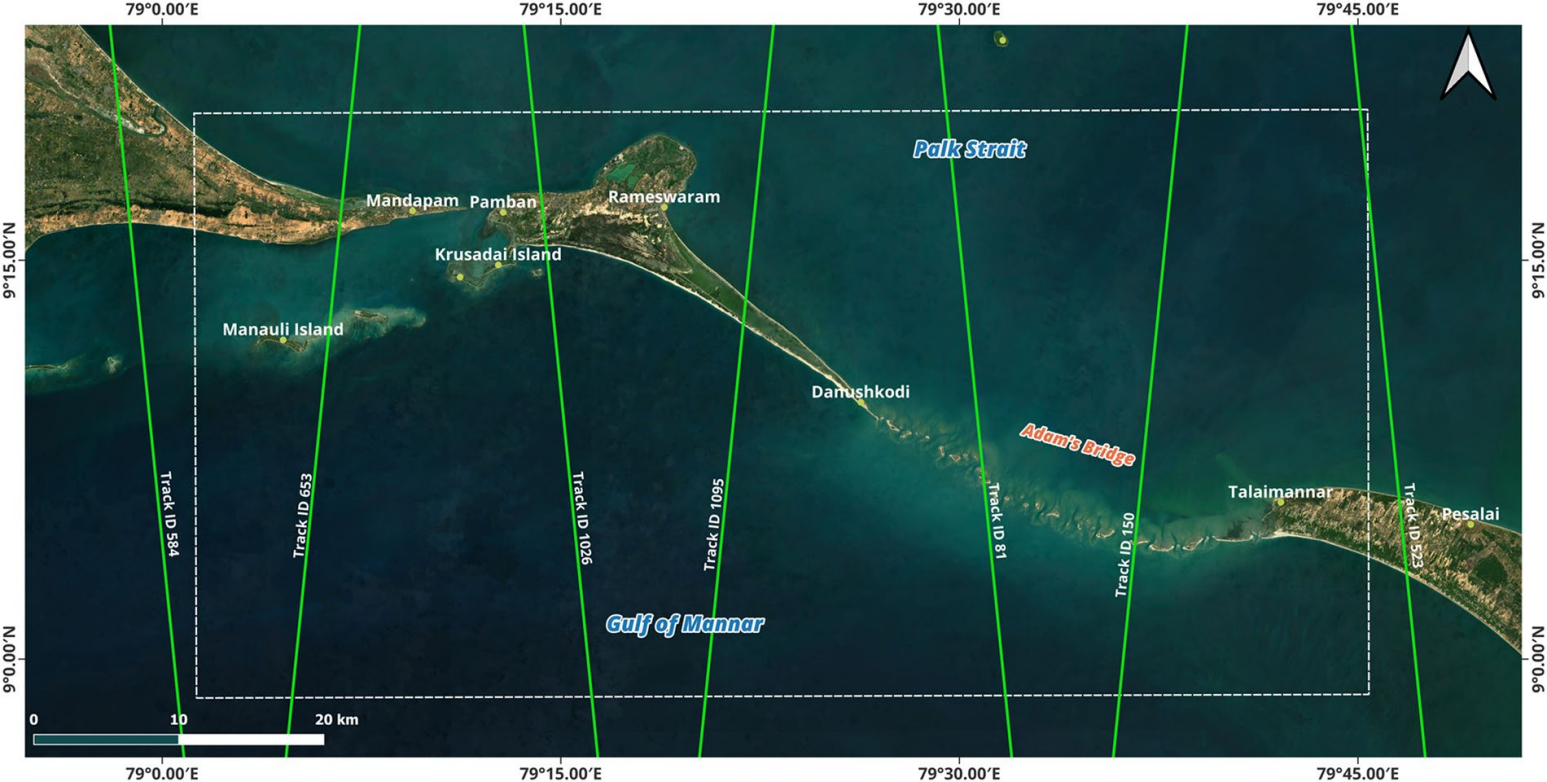
Available reference ground tracks of ICESat-2 mission over the extent of Adams’ Bridge. The satellite imagery used in the map is from web mapping services of the Sentinel-2 cloudless layer for 2021 by EOX (https://s2maps.eu/ and https://esa.maps.eox.at/), and the source of ICESat-2 ground tracks is https://icesat-2.gsfc.nasa.gov/science/specs.

The primary data resource used to generate DBEM for Adam’s Bridge is the depth information of the seafloor retrieved using water-penetrated ICESat-2 photons. The ICESat-2 mission operates in a non-sun-synchronous orbit (thus, both the day-time and night-time acquisitions are possible) from an average altitude of 496 km with a temporal resolution of ~91 days, during which 1387 unique ground track acquisitions will be possible for the global extent^73^. The along-track sampling interval of ICESat-2 photons is 0.7 m (spatial resolution). For the extent of Adam’s Bridge, there exist seven ICESat-2 ground tracks (shown in Fig. 2), and thus, data acquisitions from these available ground tracks were considered in this research. From these seven reference ground tracks, one hundred thirty-three tracks of ICESat-2 data acquisitions were available between October 2018 and October 2023 for the study area considered in this research.

**Fig. 3:**
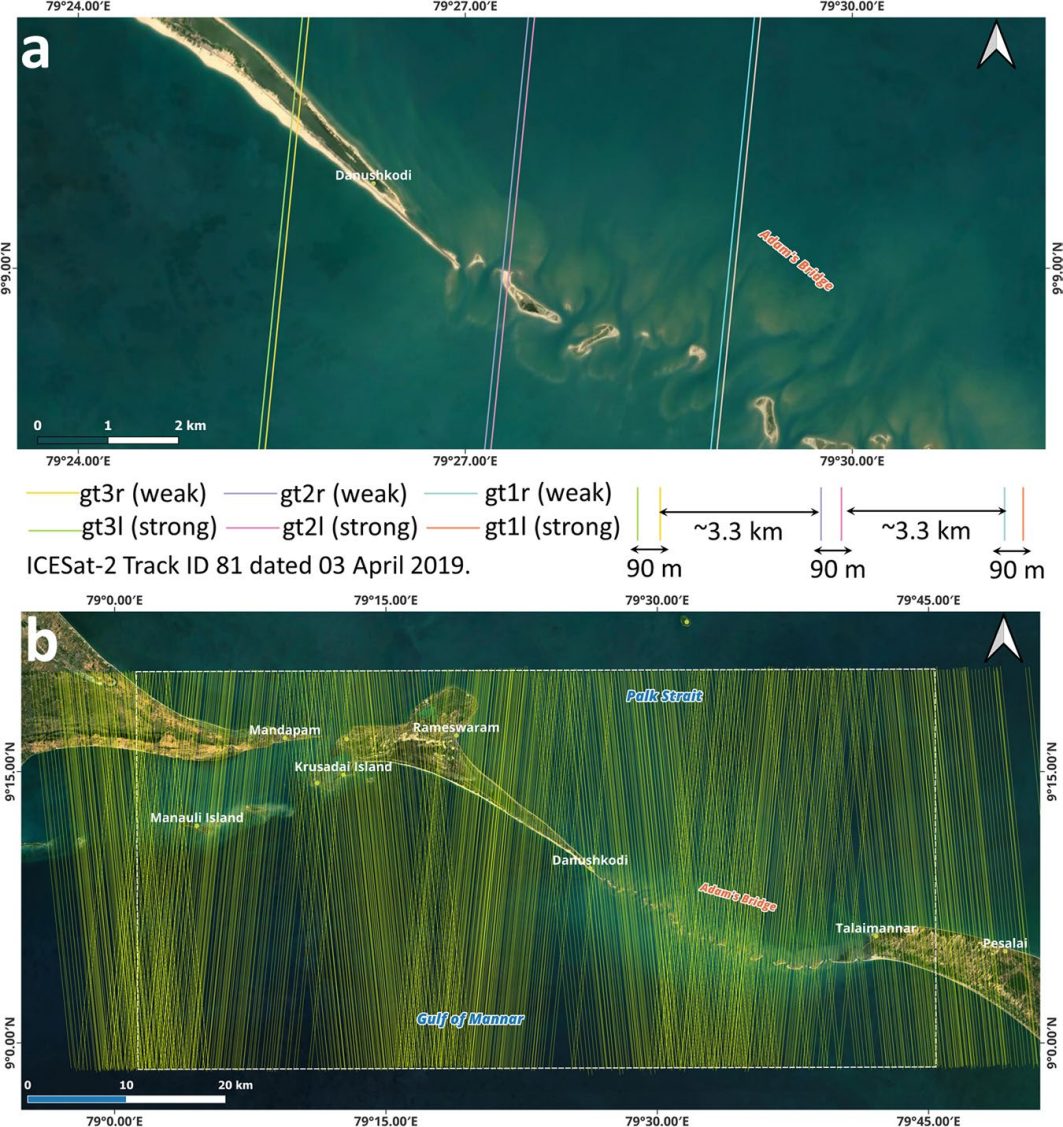
Beams of ICESat-2 acquisitions. (**a**) Six beam configuration of ICESat-2 organized as three pairs – left, near-nadir, and right. Within each pair, one strong beam and a weak beam have a separation of 90 m between them. The left and right paired beams are 3.3 km apart from the nadir most paired beam. (**b**) Qualified beams (both strong and weak) over the extent of Adam’s Bridge used to generate a digital bathymetric elevation model.

ICESat-2, equipped with a solo sensor called Advanced Topographic Laser Altimeter System (ATLAS), uses a 532 nm wavelength (green) laser operating at a pulse repetition frequency of 10 kHz. A single incident of laser from ATLAS encounters a diffractive optical element before splitting into six beams (organized as three pairs – left, near-nadir, and right); of which, within each pair, one beam (termed as a strong beam) has four times the energy of the other (termed as a weak beam) with a separation of 90 m between them^73^. Figure 3a illustrates the concept of ICESat-2’s multi-beam data acquisition over a part of Adam’s Bridge. The left and right paired beams are 3.3 km apart from the nadir most paired beam.

Once the laser beams from ICESat-2 hit the earth’s surface, reflected photons recorded by the photon-counting telescope mounted on the ATLAS sensor yield the range measurements. Data related to the ICESat-2 platform (like position, orientation, attitude, and orbital velocity), laser pointing vectors, pulse emission timing, and the range measurements will be assimilated to generate the geodetic position (latitude and longitude) and ellipsoidal heights for each geolocated photon^73^. At ground stations, science teams of ICESat-2 will process and distribute various levels of data products through a web portal at https://nsidc.org/data/icesat-2 maintained by the National Snow and Ice Data Centre (NSIDC). Global geolocated photon data available as a Level-2A product under the nomenclature ATL03 contain heights above the WGS84 ellipsoid (ITRF2014 reference frame), geodetic latitude and longitude, and other relevant attributes for all the photons downlinked by the ATLAS instrument onboard the ICESat-2^81^.

For our research, ATL03 data was downloaded using the OpenAltimetry application^82^, which is available at https://openaltimetry.earthdatacloud.nasa.gov/data/icesat2/. The OpenAltimetry application, a NASA-funded collaboration between NSIDC, Scripps Institution of Oceanography, the EarthScope Consortium, and the University of California San Diego, is a web-based cyberinfrastructure platform that allows users to locate, visualize, and download ICESat-2 surface elevation data and photon clouds for any location on Earth^82^. Photon data acquisitions from the ICESat-2 are available in Comma Separated Values (CSV) format; thus, the data can be analyzed in any electronic Spreadsheet application like Microsoft Excel or Google Sheets. Even though ATL03 is a non-imaging product (tabular data), the presence of attributes like geodetic latitude and longitude in the data will enable us to infer and analyze in the spatial domain using Geographic Information System (GIS) software like ESRI ArcGIS (https://www.esri.com/) or QGIS (https://www.qgis.org/).

**Fig. 4:**
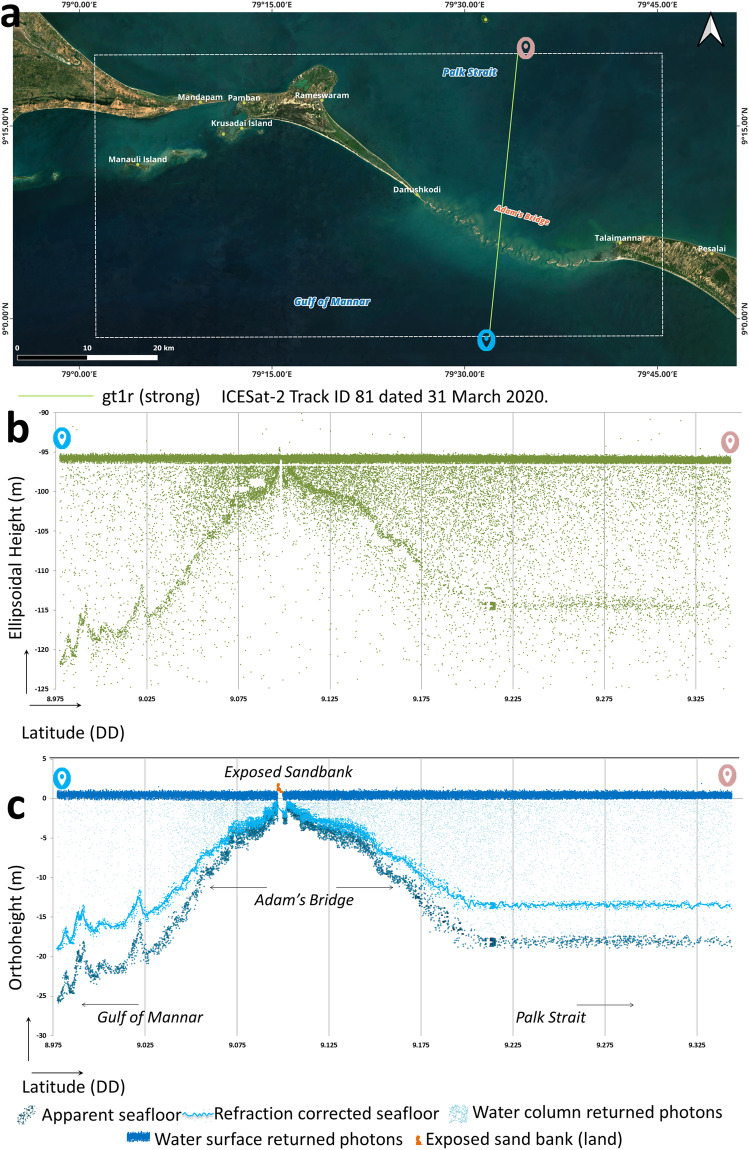
Illustration of ICESat-2’s data acquisition over water bodies. (**a**) A subset of ICESat-2 single beam acquired over the Adam’s Bridge. (**b**) The two-dimensional profile generated from the ICESat-2 photons for an extent of Adam’s Bridge - primarily, the photons were returned from the water surface, water column, and seafloor. A small patch of exposed sandbank is also evident from the profile. (**c**) 2-dimensional profile showing classified photons. Differences in depths computed from the photons of apparent seafloor and refraction-corrected seafloors can be perceived.

Along-track 2D profiles generated from a sequence of ICESat-2 photon data enable visualization of Earth’s surface variations. Figure 4a shows a subset of ICESat-2 geolocated photons overlaid on high-resolution satellite imagery for the extent of the study area. Figure 4b shows the accumulated geolocated photons in the 2D profile chart, with the X-axis having the along-track latitude acquired by the ICESat-2 ground track and the Y-axis having the elevation (ellipsoidal height) in meters.

During ICESat-2’s data acquisition over the water bodies, most photons will get reflected from the water surface. However, depending on the optical properties of the water, some of the photons will return from the water column and seafloor^76–78,83–85^ (shown in Fig. 4b). The diffuse attenuation coefficient for downwelling irradiance, K_d_(λ) (in m^−1^), where λ is the wavelength of light, is a measure of how light dissipates with depth in water; it indicates how strongly light intensity at a specified wavelength is attenuated within the water column and generally computed from remote sensing techniques^86,87^. Open access and daily temporal resolution-based data like K_d_(490) is available from the Sentinel-3 A/B OLCI level-2 series of data product services and helps characterize the transparency of water^88–90^. For successful seafloor detection up to a depth of ~40 m from the ICESat-2 photons, the recommended value^76,91^ of K_d_(490) should be less than 0.12 m^−1^. In our research, acquisitions during high turbid load in the water were avoided by referring to the K_d_(490) of Sentinel-3 A/B; during this crosschecking procedure, the overlap period between ICESat-2 and Sentinel-3 A/B acquisitions is within +/− 24 hours. Similarly, for water depth-related studies using ICESat-2, it is recommended to prefer night-time acquisitions because the background noise caused by solar spectral radiation significantly impacts the seafloor detection performance of LiDAR^92,93^. Thus, only those acquisitions of ICESat-2 obtained during night-time were considered to generate DBEM for the study area.

Abiding by prerequisite conditions like preferring night-time acquisitions and omitting the data acquired during turbid load periods, 66 tracks are found qualified from the available 133 tracks, and these tracks comprise 396 strong and weak beams of along-track data. All these 396 beams (shown in Fig. 3b) of data were processed to classify the returned photons from the water surface, water column, land, and seafloor using Density-Based Spatial Clustering of Applications with Noise (DBSCAN)^94–97^ followed by manual correction using localized statistical algorithms^98,99^ to eliminate the outliers. Typically, the DBSCAN algorithm, by taking parameters like radius and threshold of minimum points, will make clusters of points as individual units/classes within the said radius when the density of points exceeds the pre-set threshold of minimum points. Also, the DBSCAN algorithm identifies the points as outliers in low-density regions (refer to Fig. 4b,where outliers exist above the water surface and below the seabed). Figure 4c shows the classified photons based on their interaction with the surface feature for a subset of the ICESat-2 beam.

The default vertical datum represented by the geolocated photons in the ICESat-2 ATL03 data product is WGS84 ellipsoid^81^, which needs to be converted to orthometric heights during the bathymetric estimations^100,101^. While generating the DBEM for Adam’s Bridge, we used the EGM2008 geoid model and geoid height calculator at https://www.unavco.org^102^. By default, photons that have returned from the seafloor are apparent and need applying refraction correction to retrieve the actual depth^76^; this is because there will be a change in the speed of light that occurs at the air-water interface due to the difference of refractive indices of air and sea-water. Avoiding the refraction correction for the depths from the photons returned from the seafloor includes vertical errors in the bathymetric estimation due to apparent depth values. Figure 4c shows the difference in depths due to the apparent seafloor and the refraction-corrected seafloor. For the DBEM generated for the extent of Adam’s Bridge, we implemented the refraction correction method^76,85^ based on Eq. 1.

1 $${Dept}{h}_{C}={Dept}{h}_{{apparent}}\left(\frac{{n}_{1}}{{n}_{2}}\right)$$

In Eq. 1, Depth_C_ is the refraction-corrected depth of the seafloor, and Depth_apparent_ is the apparent depth (default depth) of the photons returned from the seafloor. n_1_ ≈ 1.00029 and n2 ≈ 1.34116 are the refractive indices of air and water bodies, respectively.

**Fig. 5:**
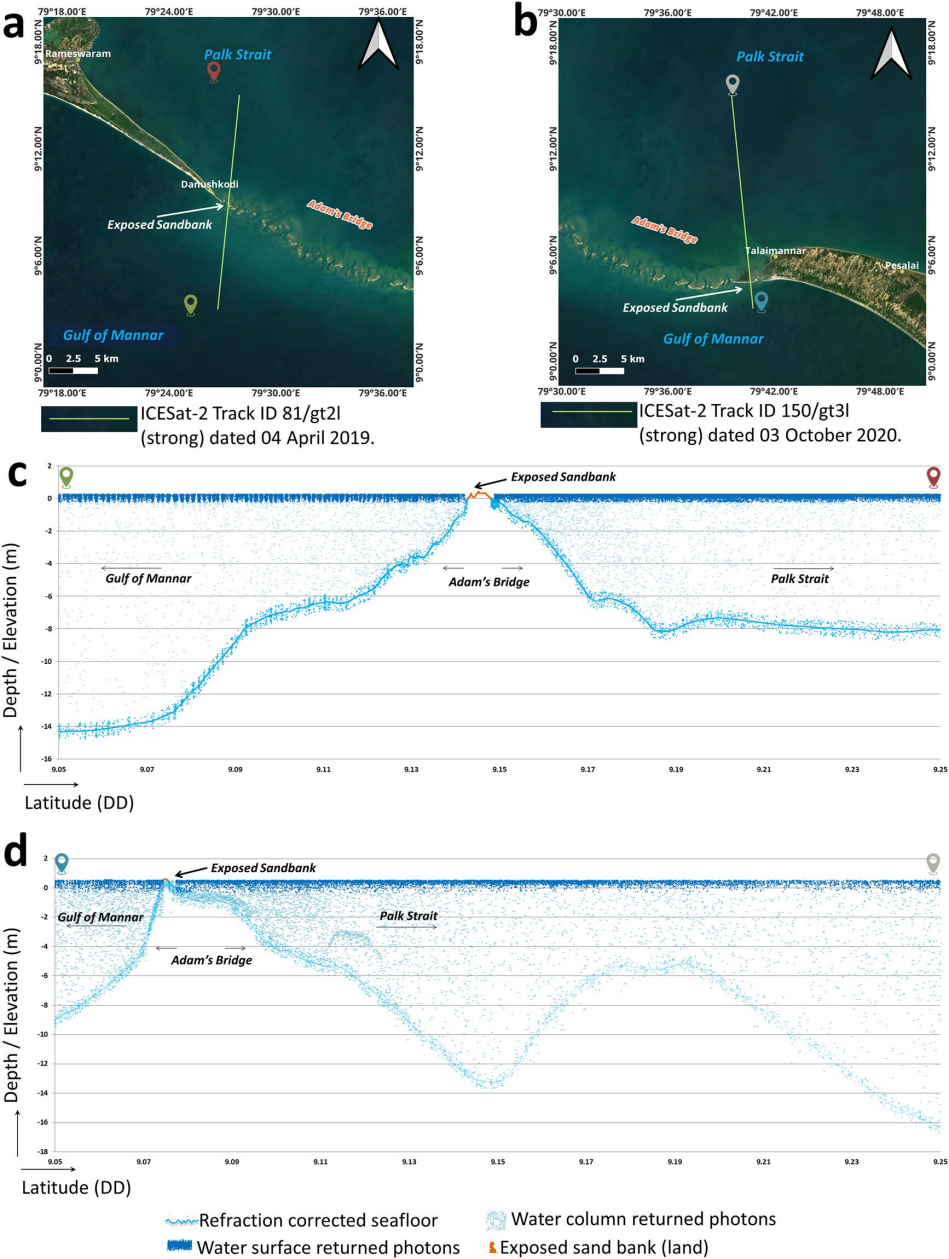
Illustration of seafloor variation at Adam’s Bridge using the profiles from ICESat-2 photons. (**a**) ICESat-2 beam acquired over the Adam’s Bridge (at the tail-end of Dhanushkodi) and overlaid on the satellite data. (**b**) Subset of the ICESat-2 beam acquired over the Adam’s Bridge (at the head of Talai Mannar) and overlaid on the satellite data. (**c**) 2-dimensional profile showing the refraction-corrected seafloor for the beam corresponding to (**a**). (**d**) 2-dimensional profile showing the seafloor variation for the beam corresponding to (**b**). Both the profiles shown in (**c**) and (**d**) signify the presence of Adam’s Bridge’s structure, which gradually rises from a depth of nearly 8 m to the surface of the water level.

Only those photons that returned from the seafloor and land were considered for further processing. Photons returned from the water surface and water column were discarded during the computation of bathymetry as they do not have any role in estimating the seafloor depths. Figure 5 shows profiles generated for two different acquisitions of ICESat-2 beams at the extent of Adam’s Bridge. It is evident that from Fig. 5c,d, both the exposed sandbanks (above the mean sea level) and the seafloor depths are seen in the profiles; thus, to understand the intricate details of Adam’s Bridge, elevation values (of those features that are above the mean sea level) and the sea floor depth prove to be vital; else, a simple digital bathymetric model that holds only depth values (below the mean sea level) may not give information about the exposed features of the study area.

**Fig. 6:**
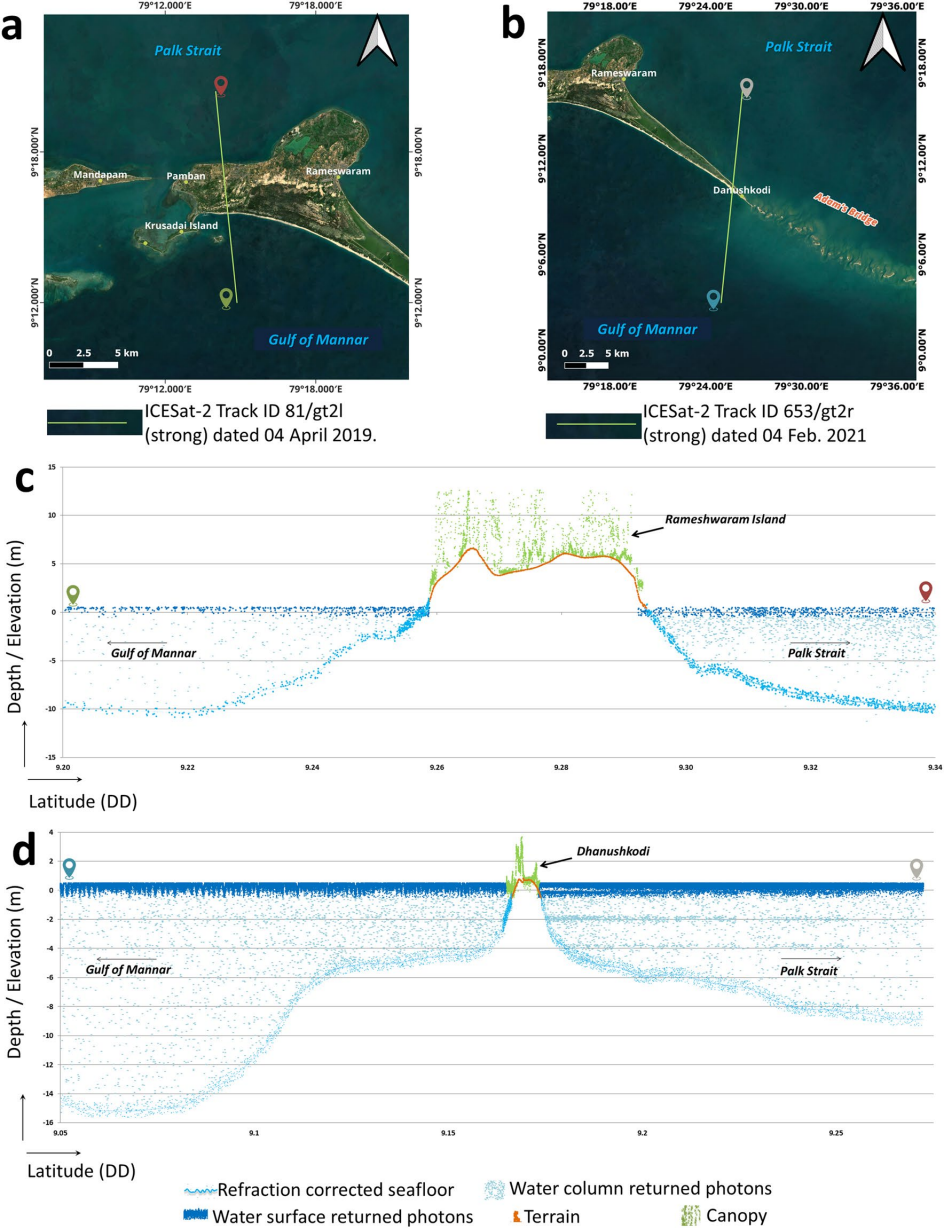
Illustration of ICESat-2 photons acquired over the extent of land and seawater surrounding Adam’s Bridge. (**a**) ICESat-2 beam acquired over Rameshwaram Island and overlaid on the satellite data. (**b**) ICESat-2 beam acquired over Dhanushkodi and overlaid on the satellite data. (**c**) 2-dimensional profile showing the seafloor and the land/canopy for the beam shown in (**a**). (**d**) 2-dimensional profile showing the seafloor and the exposed land part at the Dhanushkodi with respect to the beam shown in (**b**).

Figure 6 shows the profiles for two acquisitions of ICESat-2 beams over Pamban Island and Dhanushkodi, which include the land elevation and the seafloor depth. In general, depending on the surface features over the land, ICESat-2 photons may return from the bare earth as well as from the canopy/built-up features^85,103,104^. Generally, photons returning from the ground are denser in the horizontal direction than in the vertical direction. In contrast, the photons returning from the canopy have a high density in the vertical direction (refer to Fig. 6c). Using this as a hint, algorithms like DBSCAN can easily distinguish the photons returning from the ground and canopy^105,106^. In this research, we have classified the photons returned from the ground and surface features using DBSCAN, and finally, towards generating the DBEM for the study area, only those photons that returned from the bare earth were considered at the land parts, and the photons falling on the canopy/built-up were discarded (refer to Fig. 6c,d).

Approximately 0.2 million ICESat-2 photons representing the values of the seafloor depths and land elevations were collected as a part of the data aggregation process; from this, a few well-distributed points were reserved as checkpoints for technical validation of the output (discussed in the subsequent sections). To generate a continuous surface, the point database containing the accumulated depths and the land elevation values from the ICESat-2 photons must undergo interpolation^107^. Interpolation is a mathematical process to predict unknown values using the surrounding measured values^108^. However, the pattern of data collection through ICESat-2 is along-track, and solely using the along-track points in the interpolation methods may not yield a continuous surface as gaps between individual tracks impact the point distribution. The maximum gap in the study area is ~400 m in our collection of along-track points. Thus, additional points are needed to compensate for the fill-gap issue. For this, ~4.9 thousand points of depth values were extracted from available Electronic Navigation Charts (ENCs)^109,110^ mentioned in Table 1. The available ENCs for the study area accord with the International Hydrographic Organization (IHO) standard called S-57, which is a data transfer standard used for the exchange of digital hydrographic data between national hydrographic offices and its distribution to manufacturers, mariners, and other data users^111^. These ENCs are processed using the tools available in ESRI’s ArcGIS software^112^. Earlier, the procedure for using ENCs in GIS platforms was described by Hui *et al*.^113^ and Taylor *et al*.^114^. The procedure to retrieve the sounding depth values and the associated geodetic coordinates from the ENCs includes reading the values from a data layer titled Geographic Reference Record. During the retrieval of depth values from the ENCs, it was ensured, especially for the gap areas, that a minimum of 2 points were retrieved successfully. A few points from the pool of sounding depths acquired from the ENCs were reserved as checkpoints for technical validation.

**Table 1:** Details of the data sources used to generate a digital bathymetric elevation model for the Adam’s Bridge.

Data source	Remarks/Usage	Details of data availability
ICESat-2 ATL03	• Ground tracks: 584, 653, 1026, 1095, 81, 150, and 523. • Preferred acquisitions: January to May (low turbid period) and mostly night-time acquisitions. • Refraction-corrected photons returned from the seafloor were used to compute the bathymetry. • Elevations from the bare earth returned photons were used to estimate the digital elevation model for the land part. • ICESat-2 Approximately 0.2 million ICESat-2 photons representing the seafloor’s depths and terrain elevations were collected as a part of data collection.	https://nsidc.org/data/icesat-2 or https://openaltimetry.earthdatacloud.nasa.gov/data/
Sentinel-3A/B	• Correspondingly, for the exact dates acquired by the ICESat-2, within +/− 24 hours, Level-2 OLCI data products from the Sentinel-3 A/B mission were used to retrieve K_d_(490). • Kd(490) data is used to assess the turbid load in the study area. Only those acquisitions of ICESat-2 were considered while K_d_(490) < 0.12 m^−1^, i.e., clear water conditions.	https://sentinels.copernicus.eu
Sounding depths from Electronic Navigational Charts (ENCs)	To increase the density of points representing the seafloor’s depth in the study area, sounding depths were digitized from the following ENCs datasets ENC No: IN2262AB at Scale: 300000 ENC No: IN3316AA at Scale: 150000 ENC No: IN53016M at Scale: 37500 Chart No: 3016 at Scale: 37500 Chart No: 3040 at Scale: 50000 These charts are issued by hydrographic offices, as per the International Hydrographic Organisation’s (IHO) standards, specifications, and symbol sets.	https://hydrobharat.gov.inhttps://iho.int
Forest And Buildings removed Copernicus DEM (FABDEM)	Towards densification of elevation points for the extent of the area having the land part (like Rameshwaram and Talai Mannar Islands), elevation values from FABDEM were used.	https://data.bris.ac.uk

**Fig. 7:**
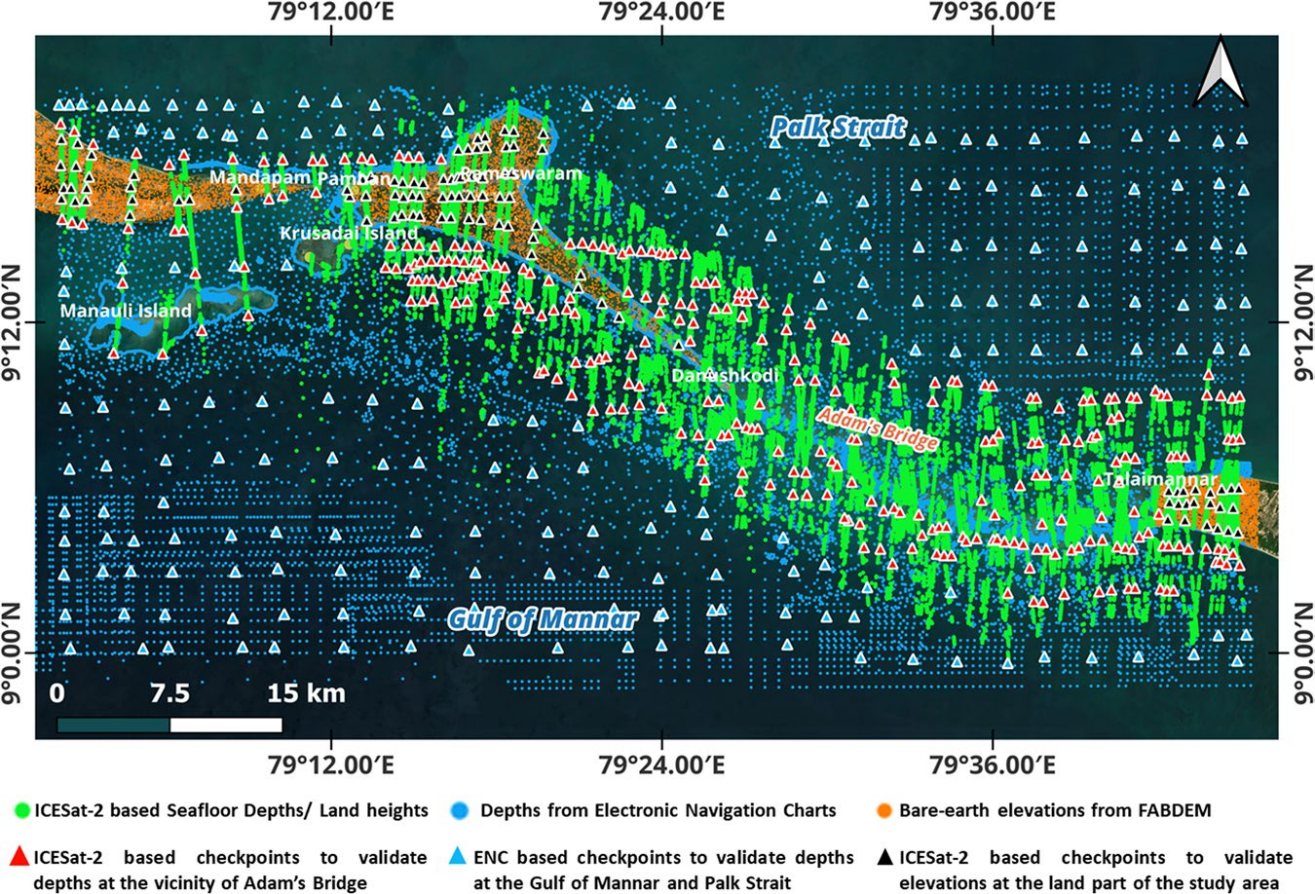
Map showing the distribution of points representing the seafloor depths and land elevations over the Adam’s Bridge and its surroundings. Nearly 0.2 million points were accrued towards creating a database primarily acquired from the ICESat-2 geolocated photons returned from the seafloor and land, sounding depths from ENCs, and land elevations from a digital bare-earth model, FABDEM. Few points that were reserved as checkpoints for technical validation of the output are also shown in the map.

**Fig. 8:**
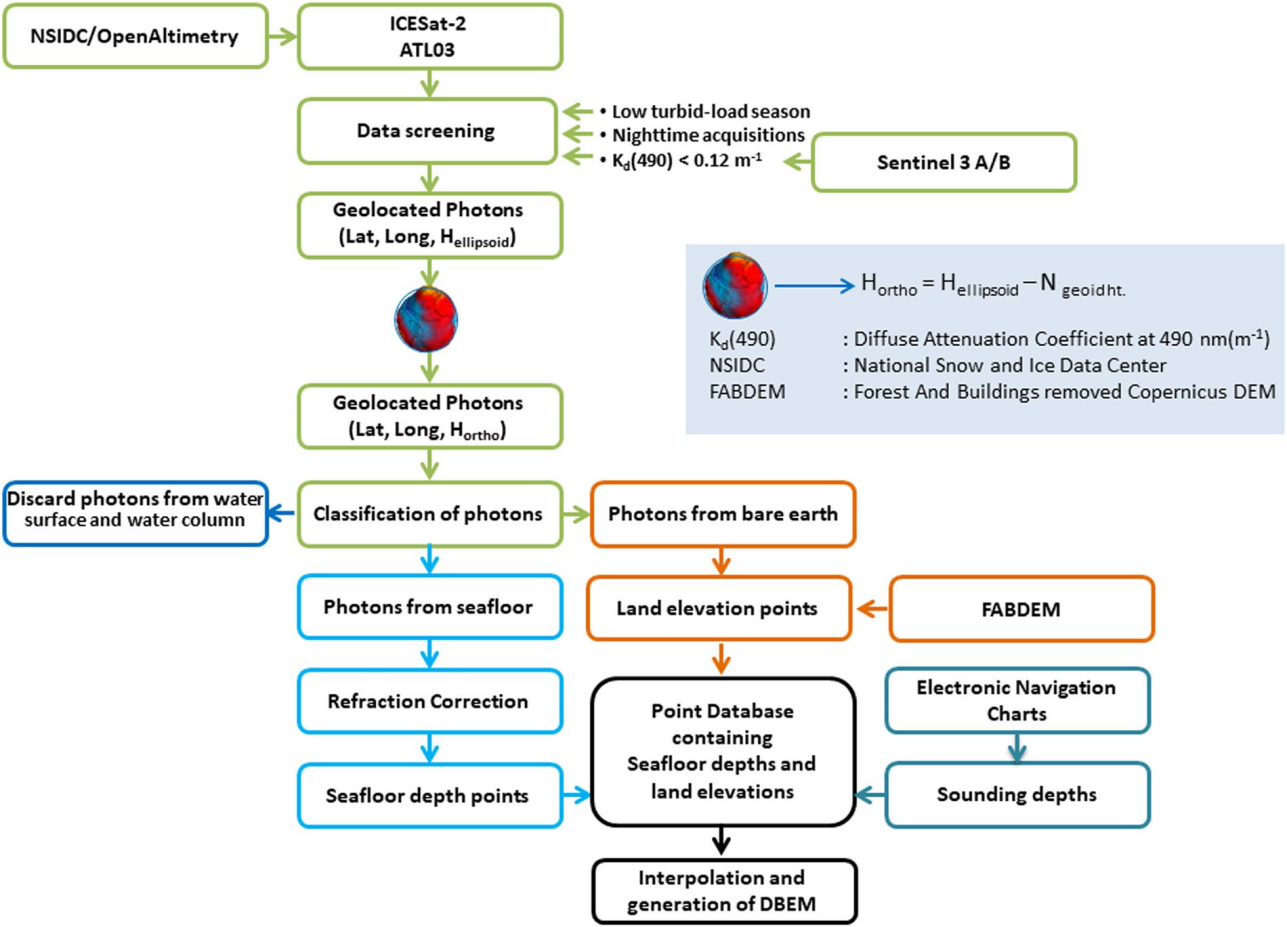
Schematic representation of methodology implemented to generate a digital bathymetric elevation model for Adam’s Bridge and its surroundings using ICESat-2 photon data.

Similarly, for the extent of having the land part (like Rameshwaram and Talai Mannar Islands), orthometric elevation values from an open-access bare-earth model called Forest And Buildings removed Copernicus DEM (FABDEM)^115^ were considered. FABDEM, available at 30 m spatial resolution, is the first kind of global DEM to represent the elevations of near bare-earth^116^. Figure 7 shows the distribution of total points considered from various sources used to generate DBEM for the study area. The details about the data sources are mentioned in Table 1. Figure 8 shows the schematic representation of the methodology implemented towards generating DBEM for Adam’s Bridge; the methodology is partly considered from the earlier researchers’ works^100^ that have attempted to derive bathymetry from the ICESat-2 geolocated photons.

Interpolation is essential in generating bathymetric surfaces from well-distributed points; the process enables estimating the values in areas lacking direct measurements. Most GIS software has implemented various interpolation methods^117^. However, there is no single choice in selecting the optimal interpolation technique, as most of them can significantly impact the accuracy based on the surface type, data density, and point distribution^107^. Interpolation techniques are generally classified into two categories: deterministic and geostatistical^117^. Deterministic interpolation techniques create surfaces based on measured points or mathematical formulas; methods such as Inverse Distance Weight (IDW), natural neighbour, and spline/trend fitting fall in the deterministic category. In contrast, geostatistical interpolation techniques, such as various forms of Kriging, are based on statistics and are used for more advanced prediction surface modeling that also includes some measure of the certainty or accuracy of predictions^117^. In our experiment, we generated surfaces using various methods available in the GIS software, namely, ESRI’s ArcGIS Pro 10.3 version (http://www.esri.com); the methods are listed in Table 2. Technical validation for all the surfaces generated using the available interpolation methods was performed using the reserved checkpoints described earlier. The study considered the DBEM with less Root Mean Square Error (RMSE) obtained with the IDW method. Earlier, researchers mentioned that IDW is considered a highly adaptable estimation method as it is the best to reconstruct natural surfaces given dense and well-distributed points in the study area^118,119^. As in our case, high-density and well-distributed points for the study area are available, and due to this, the IDW method generated a superior DBEM compared to other methods (refer to Table 2).

**Table 2:** Details of accuracies of the surfaces generated using various interpolation method.

Details of checkpoints used for quality assessment	Number of points	IDW	Oridinary Kriging	Simple Kriging	Universal Kriging	Emperical Bayesian Krigining	Natural neighbour	Spline	Trend
		RMSE (m) of Z.							
Checkpoints representing the depths obtained from the ICESat-2 photons near the vicinity of Adam’s Bridge	300	0.56	1.21	1.43	1.56	1.67	1.25	3.56	2.3
Checkpoints representing the depths obtained from the ENCs in the Gulf of Mannar and Palk Strait	200	0.72	1.46	1.95	2.32	2.12	1.43	4.95	2.9
Checkpoints representing the elevations obtained from the ICESat-2 photons for the extent of land parts	100	0.79	1.13	1.45	1.93	1.76	1.82	3.98	6.67

Determining the resolution (i.e., the cell/grid size) of the bathymetric elevation model depends on the density of the points used to generate the model. Attempting to generate a high-resolution surface model using less-density points will accumulate artifacts in the output; thus, the higher the density of points, the fewer the artifacts in the high-resolution surface model. Hu^120^, through his experiments, suggested a standard method for determining the cell size of the raster-based bathymetry/elevation model through Eq. 2.

2 $$s=\sqrt{A/n}$$

Where s is the estimated raster cell size, and n is the number of points in the minimum area of density (A) within the extent of the point distribution. From the point distribution map obtained in our experiment, it is observed that for every 200 sq.m, a minimum of 2 points representing seafloor depths/land elevations exist. Thus, the output pixel’s cell size was kept to 10 m during the interpolation stage based on Eq. 2.

## Data Records

The DBEM for Adam’s Bridge generated from this study is accessible at figshare^121^. The DBEM is in GeoTIFF file format with a cell size of 10 m (spatial resolution) projected in the Universal Transverse Mercator (UTM) coordinate system with the Zone 44 N [EPSG:32644]. The pixel values of the DBEM represent orthometric depth values for the extent with water and elevation values for the extent with peninsula/land/islands in meters.

## Technical Validation

Technical validation of the DBEM generated in this research was performed using qualitative and quantitative methods. Qualitative assessment of any modeled surface (like a Digital Elevation Model – DEM, Digital Surface Model – DSM, Digital Terrain Model – DTM, and DBEM) can be done using visual methods in a three-dimensional (3D) viewer, which in general will be available in satellite-based image processing or GIS software having advanced capabilities. However, the use of visual methods for quality checking depends on the expertise and experience of the human resource^122^. 3D perspective-based visual analysis of digital surface features is highly useful for perceiving intricate details of seabed topography^123^. Additionally, 3D perspective views with directional lighting effects and coloring schemes enhance the impressions of relief data^124^.

**Fig. 9:**
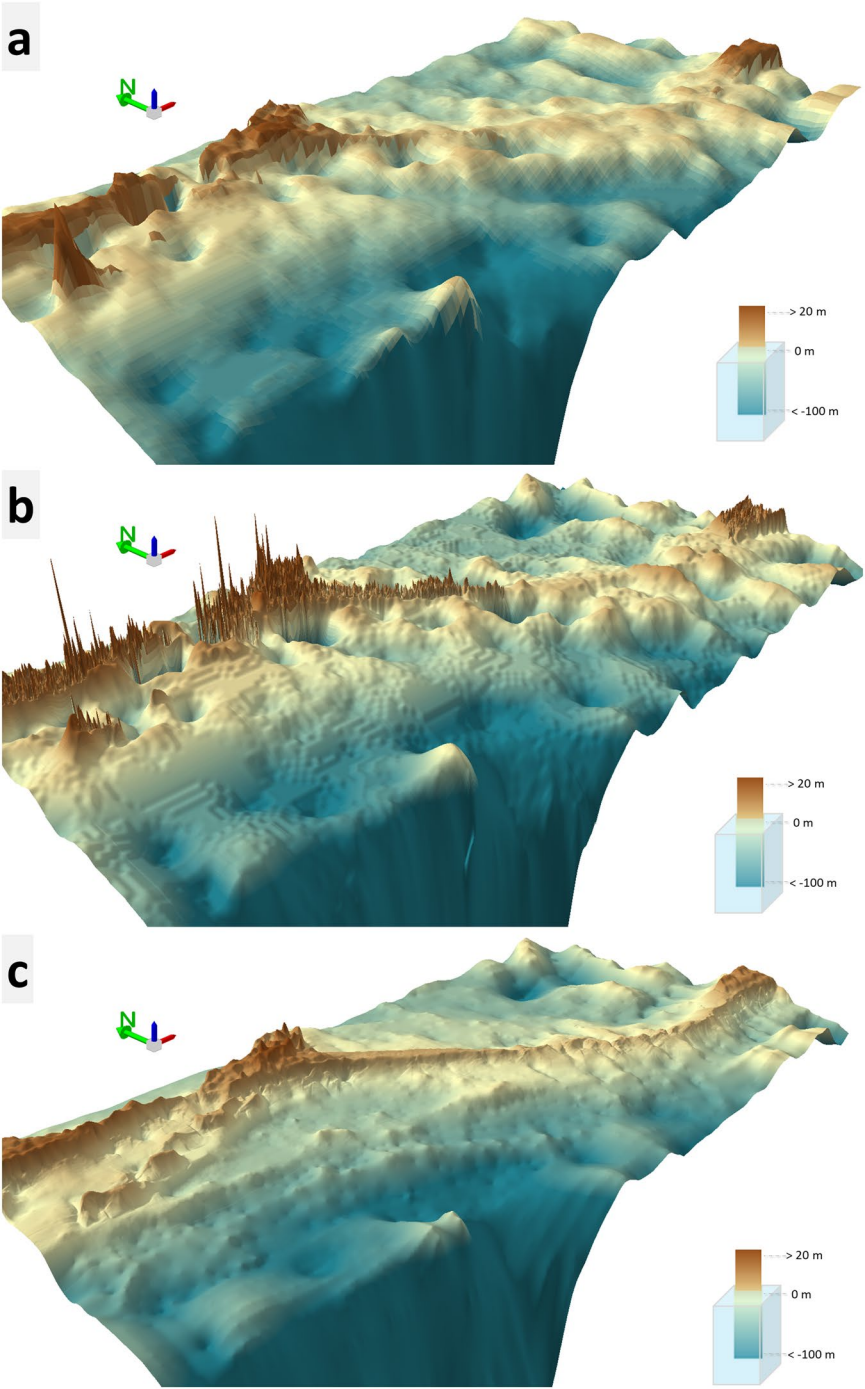
Comparison of free and open-access bathymetric data sources with the high-resolution bathymetric data generated using ICESat-2 seafloor returned photons. (**a,b**) GEBCO_2023 grid with a spatial resolution of 450 m and GMRT bathymetry with a spatial resolution of 100 m, respectively. (**c**) Digital Bathymetric Elevation data of 10 m resolution generated using ICESat-2 seafloor returned photons. These 3D perspectives views were generated using ESRI’s ArcScene Ver. 10.8.1 software (https://www.esri.com/).

During the visual analysis to assess the quality of the DBEM, it was compared with the free and open accessible global bathymetric data sources like GEBCO (latest version titled GEBCO_2023 grid) and GMRT bathymetric datasets. Figure 9 illustrates 3D perspective views of the Adam’s Bridge and its surroundings that were generated using two free and open accessible global bathymetric datasets and the DBEM generated in this research. GEBCO_2023 grid, which is of 450 m spatial resolution, exhibited a relatively flat surface, especially at the extent of Adam’s Bridge (refer to Fig. 9a); the reason may be attributed to its coarser resolution, which resulted in giving less details with respect to the seabed topography. Similarly, the digital bathymetry from GMRT, which is available at 100 m spatial resolution, not only failed to give a relief impression of the Adam’s Bridge but also accounted for artifacts in the form of spikes and large sinks (refer to Fig. 9b); the reasons for the errors can be because GMRT lacks any sounding data near the vicinity of Adam’s Bridge^72^. Visual impressions from the DBEM generated from our research with 10 m spatial resolution exhibited a higher degree of detail for the entire study area than the other two bathymetric models. Also, it has resulted in a more realistic representation of the Adam’s Bridge as a submarine continuation of Dhanushkodi and Talaimannar Island (refer to Fig. 9c). Moreover, at regular intervals of Adam’s Bridge, sudden narrow channels with depths varying between 2 to 3 m are seen which are not evident in the other bathymetric datasets. These narrow channels permit the exchange of water waves between the Gulf of Mannar and the Palk Strait. Importantly, from the crest line of Adam’s Bridge, the narrow channels are accompanied by perpendicular ridges (refer to Fig. 9c), which are nullified in the GEBCO and GMRT bathymetric datasets.

By applying the processing scheme mentioned in Fig. 8, we generated the high-resolution DBEMs for the Adam’s Bridge using various interpolation methods, in which ~0.2 million points are from the ICESat-2 photons contributing to the surface generation. During this process, 400 points of depth and elevation values from ICESat-2 photons were reserved as checkpoints towards quality checking of the output DBEMs (refer to Fig. 7). From these reserved points, 300 depth values were used to check the accuracy of the DBEM over the vicinity of Adam’s Bridge (extent having water), and 100 points were used for quality checking over the extent containing the islands. Additionally, 200 checkpoints from ENCs were used to assess the accuracy for the extent of the Gulf of Mannar and Palk Strait, where the depth is more than 40 m (refer to Fig. 7). RMSE, a statistical formula, was used to quantify the vertical accuracies for the surfaces generated using various interpolation methods and is based on Eq. 3.

3 $$\triangle H={({\rm{Depth\; or\; Elevation}})}_{{DBEM}}-{({\rm{Depth\; or\; Elevation}})}_{{\rm{checkpoints}}}$$

4 $${RMSE}=\sqrt{\frac{\sum {\triangle H}^{2}}{n}}$$

In Eq. 3, (Depth or Elevation)_DBEM_ is the set of depth or elevation values obtained from the modeled DBEMs and (Depth or Elevation)_checkpoints_ are the set of depth or elevation values of the reserved checkpoints. n is the number of observations. Table 2 summarizes the results with the model accuracies for the seafloor and terrains for all the surfaces generated using various interpolation methods. From Table 2, it is evident that the RMSE of the surface generated using IDW interpolation method performed better than all other interpolation methods with the error being less than 0.79 m over the extent of the study area.

## Usage Notes

**Fig. 10:**
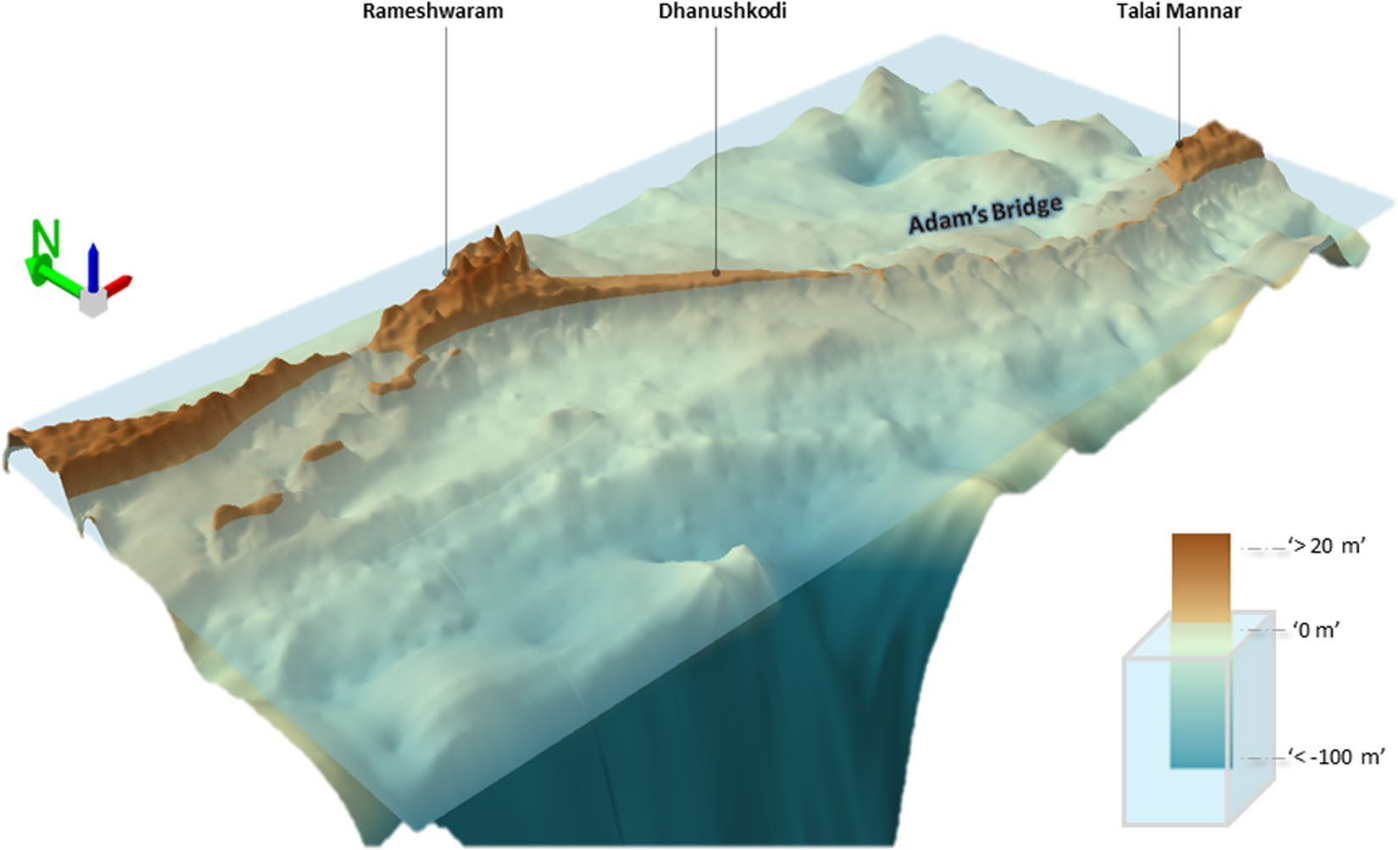
A 10 m Digital Bathymetric Elevation Model generated using ICESat-2 photons for the extent of Adam’s Bridge and its surroundings. This 3D perspective view was generated using the ArcScene module of ESRI ArcGIS Ver. 10.8.1 software (https://www.esri.com/).

**Fig. 11:**
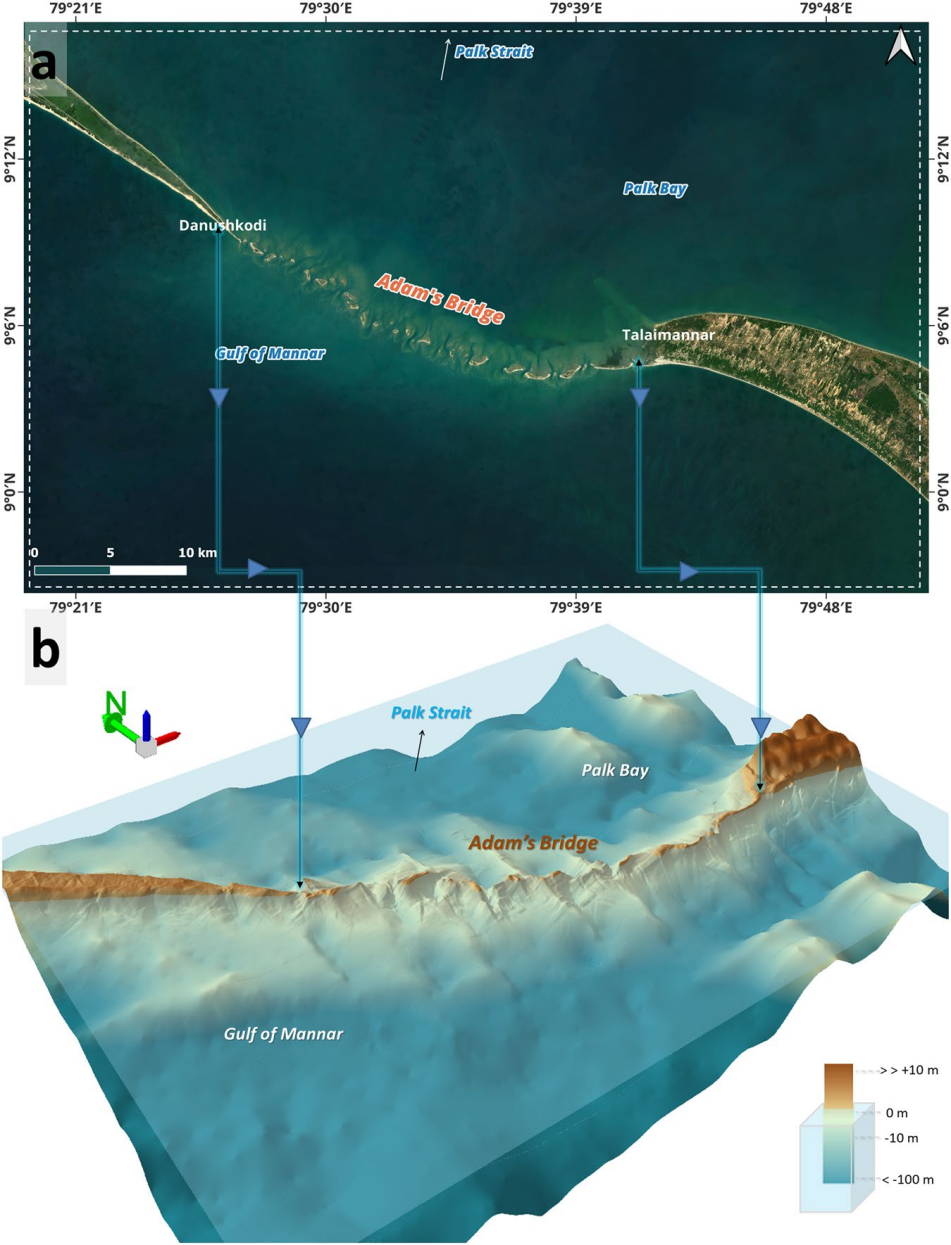
3D perspective generated with a 10 m Digital Bathymetric Elevation Model (DBEM) generated using ICESat-2 photons for the extent of Adam’s Bridge. (**a**) Extent showing the Adam’s Bridge in high-resolution satellite imagery. The satellite imagery used in the map is from the web mapping services of the Sentinel-2 cloudless layer for 2021 by EOX (https://s2maps.eu/ and https://esa.maps.eox.at/). (**b**) Perspective view for the extent of Adam’s Bridge and its surroundings. This 3D perspective view was generated using the ArcScene module of ESRI ArcGIS Ver. 10.8.1 software (https://www.esri.com/).

**Fig. 12:**
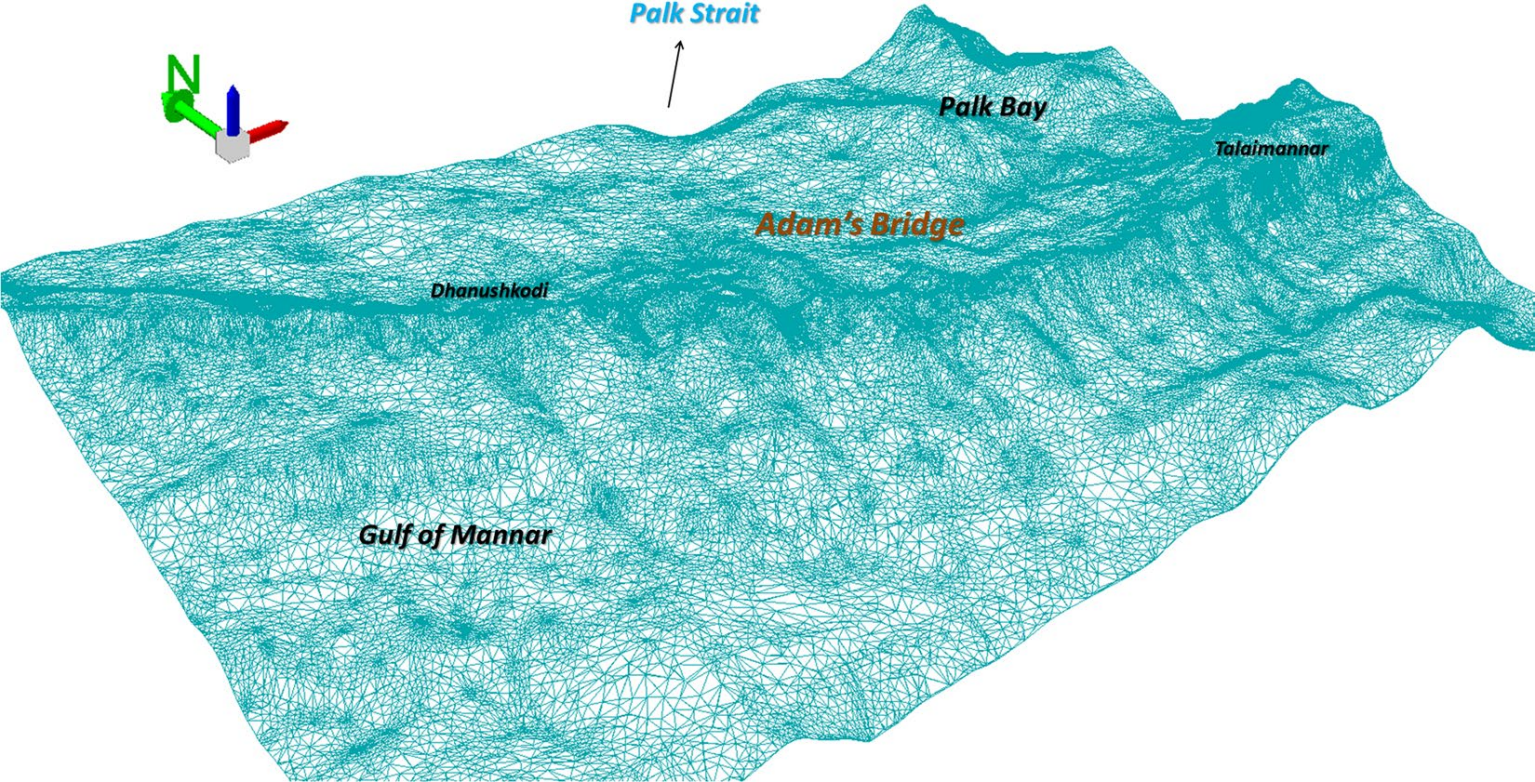
Wire-mesh mode of the 10 m Digital Bathymetric Elevation Model generated using ICESat-2 photons for the extent of Adam’s Bridge and its surroundings. This 3D perspective view was generated using the ArcScene module of ESRI ArcGIS Ver. 10.8.1 software (https://www.esri.com/).

This dataset, available in GeoTIFF format, can be opened, visualized, and further used to derive additional terrain/surface characteristics (ex., slope, aspect, and contours.) with the help of satellite-image processing or GIS software. However, for 3D visualization, the software should be equipped to support 3D viewer. Free and open-source GIS software, namely QGIS, can be used to view this DBEM in 3D viewer. Alternatively, commercial-off-the-shelf (COTS) GIS software like ESRI ArcGIS (http://www.esri.com) can be used with advanced interactive features. Figure 10 is a typical view for the extent of Adam’s Bridge generated using ArcScene module of ESRI ArcGIS software. Figure 11 shows 2D and 3D perspective view of the Adam’s Bridge. Figure 12 shows a wire-mesh mode of this dataset for a zoomed extent of the Adam’s Bridge generated in the ArcScene module of ESRI ArcGIS software (http://www.esri.com); here, the z-exaggeration was set to 200 times during the visualization to amplify the visual intricacies of Adam’s Bridge structure. The proposed DBEM can be integrated into the computational models to understand Adam’s Bridge’s morphology, surficial-sediment characterization, and wave dynamics originating from the Gulf of Mannar and the Palk Strait.

## Data Availability

ICESat-2 photon data can be downloaded from the OpenAltimetry application, available at https://openaltimetry.earthdatacloud.nasa.gov/data/ or the web portal maintained by the National Aeronautics and Space Administration (NASA) National Snow and Ice Data Center (NSIDC) at https://nsidc.org/data/icesat-2. A Python package providing implementation of the DBSCAN algorithm over ICESat-2 geolocated photons is available at 10.6084/m9.figshare.25991248. Alternatively, one can use the Create Graph module of ArcGIS software to manually classify/remove the outliers in the tabular data consisting of geolocated photons from ICESat-2. All the maps in the manuscript were compiled using ESRI ArcGIS software. Profile diagrams were generated in the MS Excel using the ICESat-2 photon data. 3D perspective views were generated in the ArcScene module of ESRI ArcGIS ver. 10.8.1 software (https://www.esri.com/).
